# Post‐Mortem Identification and Toxicological Findings of Fluetonitazepyne and Isotonitazepyne

**DOI:** 10.1002/dta.3928

**Published:** 2025-07-10

**Authors:** Pirkko Kriikku, Anna Pelander, Antti Jylhä, Ilkka Ojanperä

**Affiliations:** ^1^ Forensic Toxicology Unit Finnish Institute for Health and Welfare (THL) Helsinki Finland; ^2^ Department of Forensic Medicine University of Helsinki Helsinki Finland

**Keywords:** drug death, nitazene, NPS, overdose, postmortem toxicology

## Abstract

Nitazepyne‐type substances, such as fluetonitazepyne and isotonitazepyne, are among the latest additions to the group of *nitazenes*—highly potent and dangerous opioids that have emerged on the illicit drug market in recent years. In early 2025, five death cases in Finland tested positive for fluetonitazepyne and one for isotonitazepyne in urine drug screening. The median (range) concentration of fluetonitazepyne in post‐mortem femoral blood was 1.7 (0.4–9.5) μg/L, and the concentration of isotonitazepyne was 1.4 μg/L. Other psychoactive substances were detected in all cases. A peak corresponding to O‐dealkylated fluetonitazepyne, the 4‐OH‐nitazepyne metabolite, was present in all fluetonitazepyne‐positive urine samples and was later used in the identification of isotonitazepyne in one fatal case. This metabolite proved useful as a marker compound for nitazepyne‐type benzimidazole opioids in a urine screening.

## Graphical abstract

In early 2025, five death cases in Finland tested positive for fluetonitazepyne and one for isotonitazepyne in urine drug screening. A peak corresponding to O‐dealkylated fluetonitazepyne, the 4‐OH‐nitazepyne metabolite, was present in all fluetonitazepyne‐positive urine samples and was later used in the identification of isotonitazepynein one fatal case. This metabolite proved useful as a marker compound for nitazepyne‐type benzimidazole opioids in a urine screening.

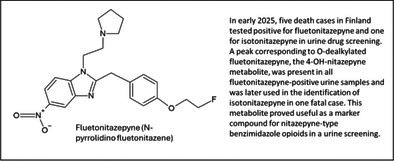

## 1 Introduction

The latest additions to the new synthetic opioid market, the 2‐benzyl benzimidazoles (*nitazenes*), are structurally distinct from earlier groups such as fentanyl analogues. Their potency has generally been shown to exceed that of morphine, making them particularly dangerous, as even small doses can cause significant and prolonged respiratory depression and death [1, 2].

Like many other new psychoactive substance (NPS) opioids, the nitazenes were originally developed by the pharmaceutical industry but were never approved for medical use [3]. The first nitazene derivative to appear in Europe was isotonitazene in 2019 [4], and since then, many countries have reported fatalities associated with nitazenes [5, 6, 7, 8, 9, 10].

**FIGURE 1:**
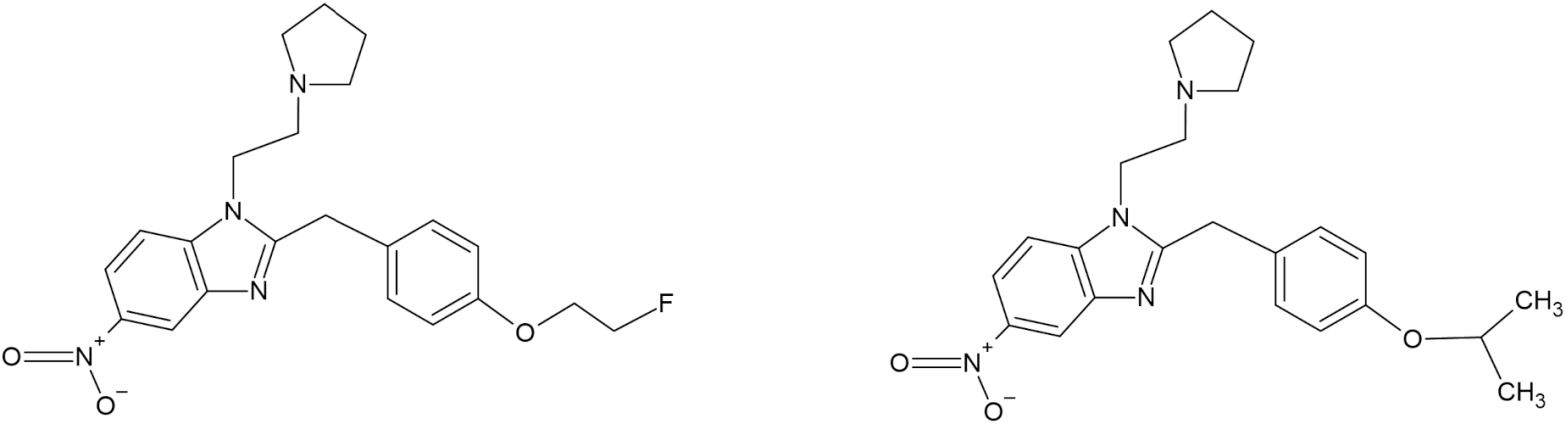
Chemical structure of (a) fluetonitazepyne (N‐pyrrolidino fluetonitazene) and (b) isotonitazepyne (N‐pyrrolidino isotonitazene).

Fluetonitazepyne or N‐pyrrolidino fluetonitazene [N‐pyrrolidino‐4′‐(2‐fluoroethoxy) nitazene] (Figure 1a) is the fluorinated analogue of etonitazepyne and was first detected in Europe in 2024 [3]. Isotonitazepyne, or N‐pyrrolidino isotonitazene [N‐pyrrolidino‐4′‐(2‐fluoroethoxy) nitazene] (Figure 1b), is another “third generation” nitazene analogue that has recently emerged on the drug market [3]. It has recently been detected in counterfeit oxycodone tablets in Australia [11]. At the time of writing, no published data exist on the pharmacology or toxicology of these substances, but preliminary findings suggest they have a potency similar to isotonitazene and protonitazene, and thus a higher potency than fentanyl [3].

Effectively responding to the threats posed by these new and potent opioids requires early recognition of emerging substances and rapid communication between authorities and the people who use drugs [12].

In this study, we report on the identification of fluetonitazepyne and isotonitazepyne in post‐mortem (PM) casework, as well as the PM blood and urine concentrations of these substances.

## 2 Material and Methods

In Finland, all sudden and unexpected deaths are subject to a medico‐legal investigation process as required by law. Most of these cases also include comprehensive PM toxicological analysis, which involves the screening and quantification of hundreds of drugs and poisons using quality‐assured methods in an accredited laboratory.

### 2.1 Chemicals and Reagents

The reference standards N‐pyrrolidino fluetonitazene citrate and N‐pyrrolidino isotonitazene citrate (Cayman Chemicals) were obtained from LGC standards. All materials and reagents used for the ultra‐high performance liquid chromatography coupled with a high‐resolution time‐of‐flight mass spectrometry (QTOFMS) screening or the liquid chromatography–tandem mass spectrometry (LC–MS/MS) analyses were of LC–MS grade purity.

### 2.2 Screening

All PM toxicology cases involving a request for drug analysis undergo comprehensive QTOFMS screening in urine or other sample matrices as described earlier [13]. In this case, after receiving information from the police regarding a suspected fluetonitazene seizure linked to a specific death, the substance was added to the QTOFMS screening database according to the “probable structure” identification rules [14], including a nitazepyne‐specific qualifier candidate (ethylpyrrolidine cleavage). A retrospective data analysis was then performed on all PM cases from the preceding 2 months.

### 2.3 Quantification

Quantification of fluetonitazepyne and isotonitazepyne in PM femoral blood and urine was performed using LC–MS/MS with multiple reaction monitoring (MRM) as described below.

Extraction: 100 μL of an internal standard mix containing metonitazene‐d3, etonitazene‐d5, and protonitazene‐d7 (0.01 μg/mL each) was added to 1 mL of whole blood and urine samples. After that, 2 mL of methanol was added to blood samples, and 2.5 mL of ethyl acetate:hexane (25:75) were added to urine samples, followed by vigorous vortex mixing and centrifuging. The organic layer was separated and evaporated to dryness, and the residue was reconstituted with a mixture of 10 μL of methanol and 90 μL of 10‐mM ammonium acetate buffer (pH 5). After centrifugation, the samples were transferred to autosampler vials.

LC–MS/MS: The LC–MS/MS instrumentation consisted of an Agilent 1295 Infinity II Pump, an autosampler, and an Agilent 6475 triple quadrupole mass spectrometer. LC separations were carried out on a Kinetex 1.7 μm EVO C18 100 Å 50 × 2.1 mm analytical column (Phenomenex) with a SecurityGuard ULTRA EVO C18 guard cartridge (Phenomenex).

The mobile phase consisted of a methanol and ammonium acetate buffer (10 mmol/L, 0.1% formic acid, pH 3.2), with the following gradient elution: methanol 20%–70% over 8 min, 70%–95% over 1 min, held isocratic for 1.5 min, then 95%–20% over 0.25 min and held isocratic for 1.5 min. The total flow rate was 250 μL/min, and the injection volume was 5 μL. The separation was carried out at 40°C.

The ion transitions of *m/z* 413.2 → *m/z* 98.1 and 153.1 (qualifier) for fluetonitazepyne, *m/z* 409.3 → *m/z* 98.1 and 56 (qualifier) for isotonitazepyne, and *m/z* 411.2 → 100.1 for isotonitazene‐d7 were monitored at collision energies of 25 and 20 eV for fluetonitazepyne and 20 and 40 eV for isotonitazepyne with a dwell time of 5 ms.

A limited fit‐for‐purpose validation was performed on this method, including precision and accuracy within and between runs over the course of 3 days. The calibration range was 0.01–100 μg/L for both analytes. Quadratic calibration was used throughout the applied concentration range with R^2^ > 0.999. The accuracy and precision of the method are shown in Table 1.

**TABLE 1:** Validation data for fluetonitazepyne and isotonitazepyne in blood and urine.

Compound	Concentration (μg/L)	Accuracy (%)^a^		Within‐day CV (%)^a^		Day‐to‐day CV (%)^b^	
		Blood	Urine	Blood	Urine	Blood	Urine
Fluetonitazepyne	0.01	91	96.5	8	9	11	11
	0.15	88.8	91.7	6	4	15	4
	1.5	91.7	98.2	2	3	3	3
Isotonitazepyne	0.01	82.6	97.8	9	5	11	8
	0.15	81.3	95.3	2	4	9	4
	1.5	87.8	98	2	4	2	4

^a^ Based on four parallel determinations (six parallel determinations at LOQ level).

^b^ Based on three separate determinations over a 3‐day period.

The limit of quantification (LOQ) for each analyte was 0.01 μg/L.

### 2.4 Research Ethics

The study was conducted under research permit THL/4850/6.02.00/2023 issued by the Finnish Institute for Health and Welfare (THL).

## 3 Results and Discussion

### 3.1 Identification and Quantitation of Fluetonitazepyne

**FIGURE 2:**
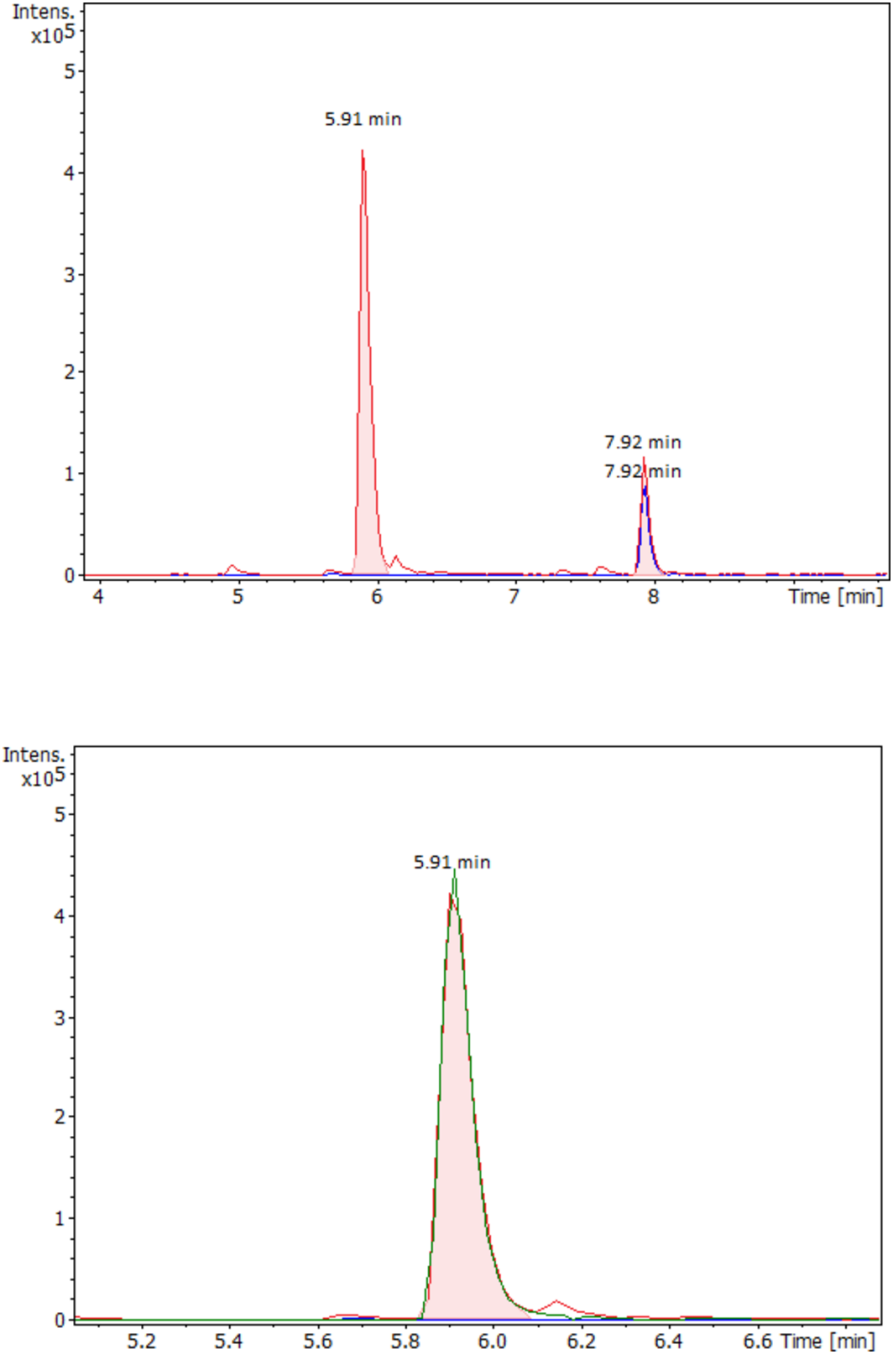
(a) QTOFMS extracted ion chromatograms in 2‐mDa mass window for fluetonitazepyne protonated molecule (*m/z* 413.1983) and qualifier candidate Q1 (ethylpyrrolidino cleavage [C6H12N]^+^, *m/z* 98.0964) strictly superimposed at 7.92 min, with a secondary Q1 signal at 5.91 min. (b) Zoomed view of 4‐hydroxy nitazepyne protonated molecule (*m/z* 367.1765) superimposed with a Q1 candidate at 5.91 min in a 2‐mDa mass window.

At the time of the initial analysis of the urine sample from the first suspected fluetonitazepyne‐positive case, the laboratory did not yet have access to a certified reference standard—only the updated QTOFMS drug screening database. A re‐processing of the QTOFMS data revealed a distinct peak at 7.92 min corresponding to the exact mass of the fluetonitazepyne protonated molecule (*m/z* 413.1983). A strictly superimposed peak for the qualifier candidate (C_6_H_12_N^+^, *m/z* 98.0964) was observed in the broadband collision‐induced dissociation (bbCID) dataset. Additionally, a secondary peak for the qualifier candidate was observed at 5.91 min, matching the sum formula C_20_H_22_N_4_O_3_, which corresponds to the molecular formula of O‐dealkylated fluetonitazepyne (Figure 2).

The 4‐OH‐nitazepyne metabolite has previously been detected in connection with metonitazepyne (N‐pyrrolidino metonitazene) and protonitazepyne (N‐pyrrolidino protonitazene) [15]. In a previous study, the 4‐OH‐nitazepyne metabolite was only found in a subset of nitazepyne‐positive cases [15]. In contrast, in our study, this metabolite was detected in all urine samples, suggesting that it is likely more abundant in urine than in blood. Therefore, the metabolite may serve as a useful marker for nitazepyne‐type opioids in urine screenings.

After the arrival of the certified reference material, the presence of fluetonitazepyne was confirmed in biological samples from the deceased by QTOFMS. Retrospective data analysis revealed two additional fluetonitazepyne‐positive cases, and two more were later identified during routine casework.

The five deaths involving fluetonitazepyne occurred between late December 2024 and early March 2025 in two mid‐sized towns in Southern Finland. The femoral blood concentrations of fluetonitazepyne ranged from 0.91 to 10 μg/L (median 1.3 μg/L). Case details are provided in Table 2 (S1−S5).

**TABLE 2:** PM femoral blood and urine concentrations of fluetonitazepyne and isotonitazepyne measured by LC–MS/MS in the studied cases.

Sample	Substance	Concentration (μg/L)		Other findings in femoral blood (μg/L)
		Blood	Urine	
S1	Fluetonitazepyne	1.2	0.6	7‐aminoclonazepam 55 Nordiazepam 27 Amphetamine 910 Additionally in PM urine: Alpha‐PVP, benzoylecgonine
S2	Fluetonitazepyne	1.3	3.8	Amphetamine 2000 Benzoylecgonine 50 7‐Aminoclonazepam 150 Pregabalin 7900 Additionally in PM urine: Buprenorphine
S3	Fluetonitazepyne	0.91	23	Oxycodone 40 Noroxycodone 37 7‐Aminoclonazepam 140 Diazepam 73 Nordiazepam 34 Additionally in PM urine: Amphetamine, naloxone, benzoylecgonine, THC‐COOH
S4	Fluetonitazepyne	10	> 100	(Therapeutic concentrations of antipsychotics) Additionally in PM urine: Buprenorphine
S5	Fluetonitazepyne	8.6	43	7‐Aminoclonazepam 38 Amphetamine 310 Norbuprenorphine 0.82 Additionally in PM urine: Benzoylecgonine, methamphetamine, buprenorphine, noroxycodone
S6	Isotonitazepyne	1.4	2.6	7‐Aminoclonazepam 25 Amphetamine 550 Buprenorphine 4.9 Norbuprenorphine 1.6 Pregabalin 2300 (Therapeutic concentrations of antidepressants)

### 3.2 Identification and Quantitation of Isotonitazepyne

The marker compound 4‐OH‐nitazepyne was later used to identify another nitazepyne, as isotonitazepyne was detected in the urine sample of a PM case in early May 2025. Initially, QTOFMS gave a match for the 4‐OH‐nitazepyne marker molecule only, and a secondary peak for the qualifier ion was observed. The reverse search of the precursor data set for the secondary qualifier peak retention time yielded a match for the isotonitazepyne protonated molecule. The identification was confirmed and quantified against a reference standard afterwards. The detected concentration was within the same range that was previously observed for fluetonitazepyne (Table 2, S6).

### 3.3 Background Information on the Deceased

The mean (range) age of the deceased was 33.8 (20–47) years, and 67% were male.

In a systematic review on nitazenes, the concentration range (median concentration) of isotonitazene in peripheral blood was reported to be 0.4–9.5 (1.7) μg/L, which aligns well with the concentrations observed for both fluetonitazepyne and isotonitazepyne in our cases [7]. This supports the preliminary assumption that these substances have similar potency [3].

In all cases in our study, the deceased had used other drugs of abuse or prescription medications with potential for abuse alongside fluetonitazepyne. Most commonly, benzodiazepines, amphetamines, and cocaine metabolites were detected. Unlike in many other reports, no designer benzodiazepines were found in these cases [5] although polydrug use appears to be a common phenomenon in the context of nitazene use [7, 10]. In all cases, the other findings included central nervous system sedatives, such as other opioids, benzodiazepines, or pregabalin. These substances, when administered together or in close temporal proximity to the nitazene, have likely aggravated the respiratory depression associated with the drug.

The cause of death has not yet been established for all cases presented in this paper. However, in the finalized cases, the forensic pathologist determined the cause of death to be accidental fatal poisonings by the detected nitazene, either alone or in combination with other substances. In death cases involving NPSs, for which very little information is available, determining the cause of death can be challenging, especially when other psychoactive substances are present and have likely contributed to the fatal outcome. In such cases, keeping up to date with the latest developments and published data on similar substances, as well as good cooperation between the forensic pathologist/medical examiner and the forensic toxicologists are even more important than in suspected poisonings by the more established drugs.

### 3.4 Risk Communication

Following the tentative identification of the new substance in a seizure, the police promptly issued a press release to warn people who use drugs about a new dangerous substance referred to locally as “tippa” (“drop”) in the area where the seizure occurred. A second press release later confirmed the identity of the drug and its connection to several death cases.

This information was also disseminated through the national Early Warning System (EWS) network and via an email group consisting mainly of professionals working with people who use drugs.

The most recent case involving isotonitazepyne was communicated to relevant authorities and the public by the forensic toxicology laboratory that first detected the substance. The communication included the identity of the drug, its association with a fatality, and the geographic location of the incident.

## 4 Conclusions

This paper describes the identification of fluetonitazepyne and isotonitazepyne in forensic toxicology casework and reports on their concentrations in PM femoral blood and urine. The metabolite 4‐OH‐nitazepyne, common to all nitazepyne‐type substances, proved to be a useful marker compound in urine screenings.

The femoral blood concentrations of both fluetonitazepyne and isotonitazepyne ranged from 0.91 to 10 μg/L (median 1.3 μg/L), and other psychoactive substances were detected in all cases. All cases with a confirmed cause of death were determined to be fatal poisonings involving the detected nitazene.

Since laboratories can typically only detect substances they are actively screening for, it is crucial to receive timely information from seizures involving new drugs linked to fatalities. Determining the cause of death in cases involving novel and potentially dangerous substances can be challenging due to limited pharmacological and toxicological data. This paper aims to contribute to the scarce body of knowledge on deaths related to these “third generation” nitazene opioids. The bbCID‐based QTOFMS screening approach employed in this study allowed complete retrospective data analysis and utilization of nitazepyne specific qualifiers in the preliminary identification of true unknowns. This, and similar approaches, play a key role in catching emerging nitazenes and other NPSs.

It is also essential to communicate the risks associated with newly detected substances to people who use drugs and professionals who work with them in order to support the management of the serious public health threat these substances pose.

## Conflicts of Interest

The authors declare no conflicts of interest.

## Data Availability

The data that support the findings of this study are available on request from the corresponding author. The data are not publicly available due to privacy or ethical restrictions.
